# Augmented reality in engineering education: Strategic design and evidence-based results

**DOI:** 10.1371/journal.pone.0341815

**Published:** 2026-02-03

**Authors:** Sebastián Donaire, Rodrigo Barraza, Pedro Martínez-Pagán, Marcos A. Martínez-Segura, Luis Álvarez, Lorena Yepes-Bellver, Víctor Yepes

**Affiliations:** 1 Facultad de Ingeniería, Universidad Santo Tomás, Santiago, Chile; 2 Applied Near-Surface Geophysics Research Group, Departamento de Ingeniería Minera y Civil, Universidad Politécnica de Cartagena, Cartagena, Spain; 3 Mechanics of Continuous Media and Theory of Structures Department, Universitat Politècnica de València, València, Spain; 4 Institute of Concrete Science and Technology (ICITECH), Universitat Politècnica de València, València, Spain; Universidad Autonoma de Chihuahua, MEXICO

## Abstract

The primary objective of this study is to define and empirically validate a novel procedural methodological framework, named SEBAS (Selection, Establishment, Blueprint, Application, Synthesis), for the strategic integration of Augmented Reality (AR) learning activities in higher engineering education. The research addresses the lack of specialized methodologies for using AR in the complex and technical discipline of mining engineering and evaluates the impact of the implementation on student educational outcomes. A mixed-method quasi-experimental design was employed, involving 136 undergraduate students enrolled in mining-related programs. A specific group of these students participated in augmented reality (AR) workshops developed using the SEBAS framework. Quantitative data on academic performance history were collected, along with qualitative data from pre- and post-intervention surveys to assess students’ perceptions and motivation. The results demonstrated a substantial positive impact on academic performance. Participating students achieved a higher average final grade (4.70, SD = 1.75) compared to the overall group (4.30, SD = 1.98). Welch’s t-tests confirmed statistically significant differences in the courses with higher AR integration (Course 1, t(36)=3.30, p = 0.002; Course 2, t(38)=10.03, p < 0.001). A thorough qualitative analysis was conducted, which yielded substantial evidence of a considerable positive shift in student perceptions, levels of interest, and practical applicability. The primary contribution of this research is the SEBAS framework, which offers a structured, pedagogically sound, and replicable model for incorporating immersive technologies. This framework facilitates the development of strategically designed AR activities that enhance academic performance, promote cognitive engagement, and prepare students for the challenges of the industry.

## Introduction

### Augmented reality in engineering education

Augmented reality (AR) has emerged as a transformative technology within the fields of education and STEM (Science, Technology, Engineering and Mathematics) disciplines [1]. Recent scholarly evidence indicates a sustained and substantial increase in research related to augmented reality (AR) applied to learning environments, demonstrating both a broadening of academic production and heightened scholarly visibility since 2011 [2]. A thorough review of bibliometric analyses [3–5], reveals that the peak period for research activity was between 2023 and 2024, with a particular emphasis on domains pertaining to interactive learning environments, immersive visualization, and augmented reality applications [6].

Azuma [7] deﬁnes virtual reality as a system in which the real is combined with the virtual, where the user can differentiate and interact with this system and in turn can interact in real time with 3D objects. Therefore, it deﬁnes a coexistence of the virtual in a real space, superimposing the resource or digital model in this space observed through a screen of some technological devices [8,9]. Singh et al. [10] establish for AR that the user has the ability to work and interact with a virtual object or resource designed and programmed in three dimensions (3D) while receiving information about the activities that are being carried out, implying being connected at all times with the environment in which we are working located in the world. For this reason, AR has a coexistence of reality with virtual objects or elements generated through graphic tools produced by computers and even [11], currently, it is possible to generate such elements through mobile devices such as smartphones, tablets and graphic tablets specialized in design, allowing such information to be projected in a real environment implying a more immersive experience manipulable and interactive.

Consequently, there has been a sustained and growing interest in exploring the pedagogical uses and potential benefits of immersive technologies such as Augmented Reality (AR), Virtual Reality (VR), and Mixed Reality (MR) to determine the most effective ways to implement them [12,13]. Recent research has focused on understanding how these technologies enhance cognitive engagement, promote experiential learning, and improve conceptual understanding through interactive and spatial visualization [14]. Consequently, the challenge no longer lies solely in technological adoption, but in the systematic evaluation of instructional design models, usability, cost design and measurable learning outcomes associated with their integration [15].

Augmented Reality (AR) has been successfully explored in a variety of sectors, including construction [16–18], addressing the challenges and opportunities of using AR in healthcare [19,20], in the natural sciences, and even studies evaluating how immersive technologies influence the motivation, success, and attitudes of foreign language students [21], highlighting the potential of AR to improve engagement and cognitive outcomes in educational contexts. However, there are few studies that refer to its implementation in the strictly mining field, with the closest applications being found in industrial maintenance, logistics, and mechanics.

In light of the high complexity and operational constraints that typify practical learning in mining engineering, the current paucity of applied case studies emphasizes both the necessity and the opportunity to advance innovative instructional approaches [22]. In this regard, the technology under discussion offers significant feasibility and pedagogical flexibility, providing safe, scalable, and contextually rich environments capable of replicating authentic mining operations [23]. The application of augmented reality (AR) through interactive and immersive visualization has been demonstrated to enhance learners’ spatial cognition, procedural understanding, and decision-making skills. These attributes are of particular importance in the dynamic and constantly evolving environment of mining processes. Furthermore, its adaptive capacity facilitates the simulation of complex or hazardous environments in real time across a range of educational and training contexts, thereby reducing operational risks while promoting skill acquisition and situational awareness [24]. This potential is consistent with long-term technological forecasts that suggest that, as equipment costs decline and Extended Reality (XR) capabilities expand, immersive educational experiences will become increasingly accessible, powerful, and integral to professional training [25], virtual objects or elements can be complex enough, having the ability to visualize it anywhere with the appropriate connectivity and device, allowing immediate interaction with the augmented reality system or application.

### Challenges of practical learning in mining engineering

The research aimed to define the scope of educational strategies for students in mining-related programs, particularly in areas such as extraction methods, equipment operation, and safety management. Through the development of a structured methodology, new learning activities were designed to complement traditional training in engineering education, and this process enabled the creation of a replicable methodological model that integrates Augmented Reality (AR) to enhance practical learning experiences in mining engineering. This study addresses educational innovation and learning methodologies [26], establishing guidelines for the effective planning of teaching content supported by technological tools, the analysis of emerging technologies such as Augmented Reality, Virtual Reality, and 3D printing becomes essential for promoting development, innovation, and meaningful learning within contemporary educational environments in mining education.

### Research gap and study contribution

Despite the growing enthusiasm and generally positive perceptions reported by both students [27] and instructors, bibliometric analyses indicate that research on Augmented Reality (AR) remains in an early stage of technical maturity [28,29]. Current studies conclude that although AR enhances academic performance and student motivation, most applications have yet to reach higher levels of functional sophistication and interactivity. This tendency is even more evident in the mining field [30–32], available research remains limited and is primarily oriented toward safety training, equipment visualization, or operational simulations, often lacking a strong pedagogical structure or systematic instructional design framework.

To address these gaps, this study proposes and empirically validates the SEBAS framework (Select, Establish, Blueprint, Apply, Synthesis) as a structured and transferable methodology for designing, implementing, and assessing AR-based learning activities in mining engineering education. Unlike prior work, this research integrates pedagogical design principles, technological development, and mixed-methods evaluation within authentic educational settings, providing both procedural guidance and evidence-based results. The contribution of this study lies not only in demonstrating the educational potential of AR, but in offering a replicable methodological approach that can be adapted to other engineering and applied science disciplines.

### The industry and the education model

The incorporation of Industry 4.0 technologies in the university environment marks the path towards Education 4.0, proposing an educational model that integrates innovative courses, soft skills, and learning workshops to train engineers trained for future industrial challenges. Regarding the above, it is necessary for universities to update student and teacher curricula to face the challenges of Industry 4.0, incorporating technologies such as Big Data, AI (Artiﬁcial Intelligence), Augmented Reality and Internet of Things (IoT); in this way, preparing new generations for the industrial revolution through practical applications in industry [33].

An effective change towards industry 4.0 lies in adapting and moving towards education 4.0, with virtual learning environments and augmented reality being tools with great potential to be included in the educational process and which also requires advanced implementation to adapt the human factor to the management of the technologies required to the prevailing technological advances. To this end, seeking to adapt teaching programs to train more capable professionals by providing previous experiences useful for their performance and providing them with the competencies required by the labor market [34,35].

### Augmented reality trends applied to mining education

**Fig 1:**
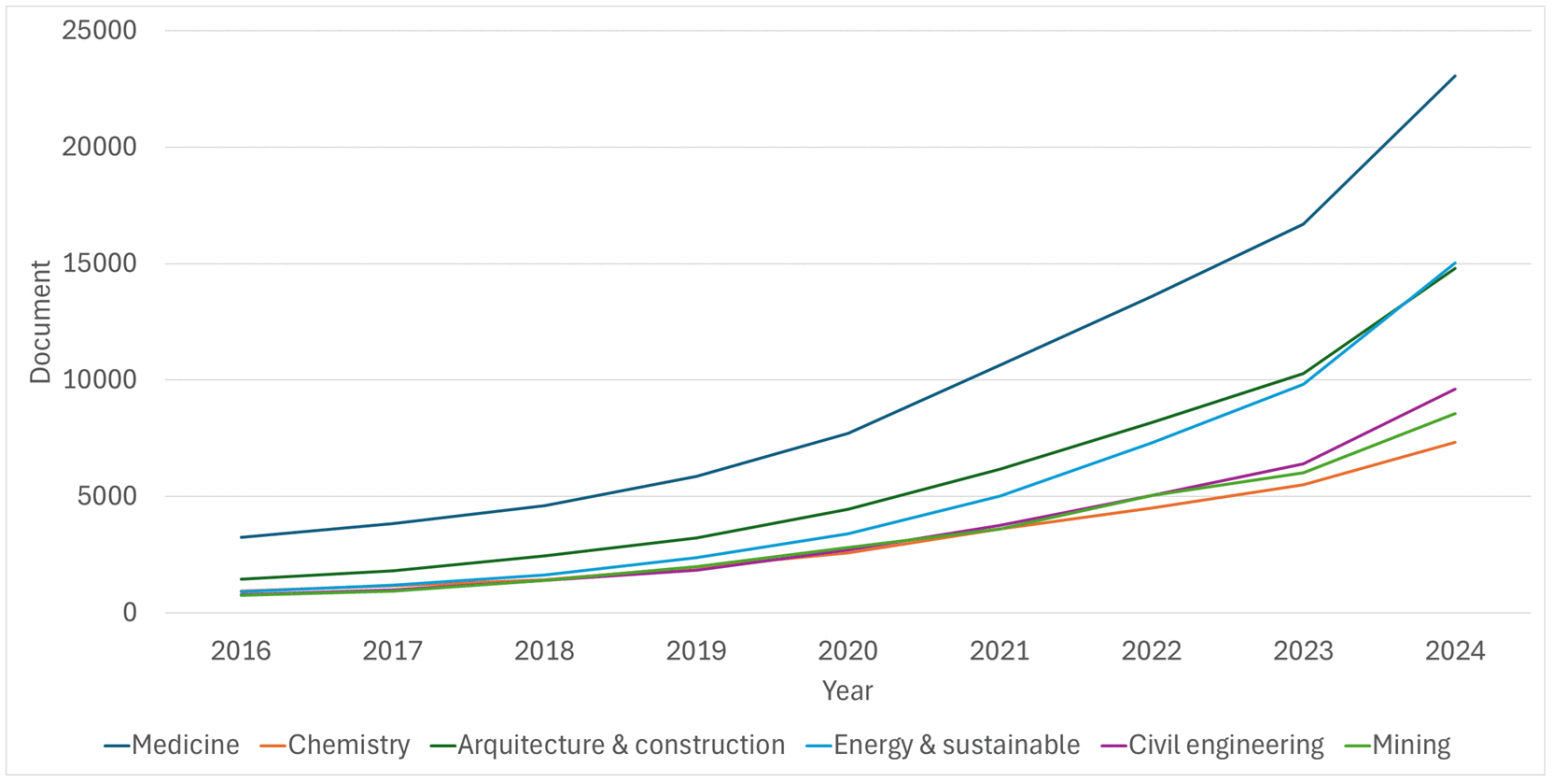
Annual distribution of Scopus-indexed publications on Augmented Reality (AR) in different disciplines (2016–2024).

The potential of these tools to revolutionize teaching in technical disciplines has been demonstrated, underscoring their value in preparing students for the specific challenges of the industry. The use of augmented reality not only provides students with advanced technical knowledge but also fosters the development of critical skills for innovation. However, despite this steady growth, the mining field remains remarkably underexplored, as illustrated in Fig 1 (Scopus analysis) and Figure Y (Web of Science analysis). A comparative analysis of augmented reality in various scientific and professional domains reveals a conspicuously lower level of application and publication in mining when compared to other disciplines such as medicine, architecture, or civil engineering.

A bibliometric analysis was conducted using the Scopus and Web of Science (WoS) databases to define the current state of research on immersive technologies in different domains of knowledge. The search strategy employed a combination of terms related to augmented reality and virtual reality, along with those denoting education or training, and keywords specific to various disciplines, including medicine, chemistry, architecture, energy, civil engineering, and mining. The analysis covered the period 2016–2025 and included only published articles from peer-reviewed scientific journals.

**Fig 2:**
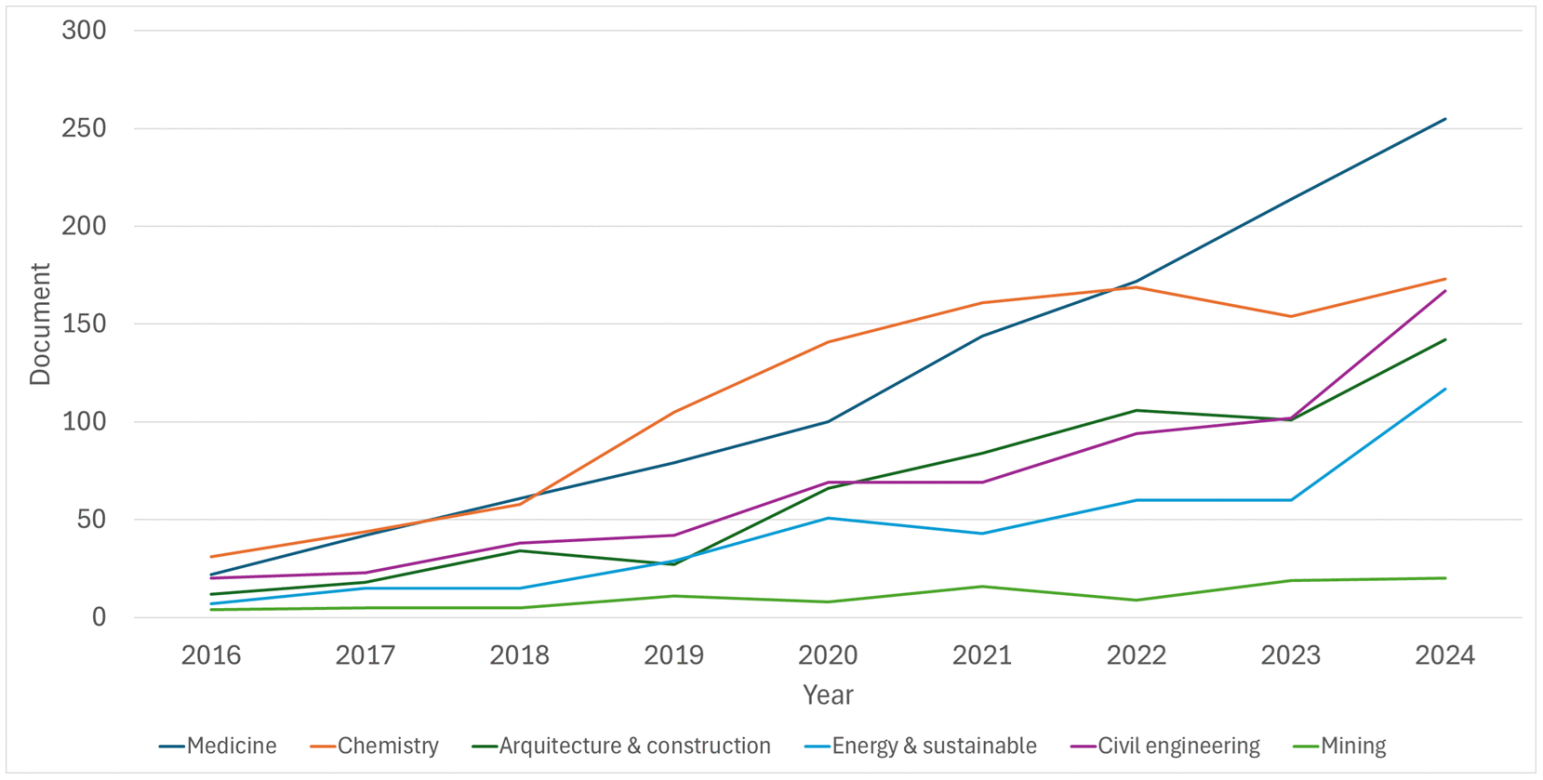
Annual distribution of Web of Science (WoS)-indexed across different disciplinary fields (2016–2024).

The WOS data presents a more selective pattern, with fewer but higher-impact studies, confirming a similar gap in mining-related applications compared to what can be seen from the Scopus analysis (Fig 2). This evident disparity highlights a significant research opportunity: the urgent need to encourage and expand the implementation of immersive and augmented reality technologies in mining engineering education and training, promoting learning environments.

## Materials and methods

The implementation of active and technology-enhanced methodologies in strategic planning is a response to generational change, digital transformation, and evolving educational paradigms in higher education [36,37]. As asserted by [38], the efficacy of educational innovation is contingent upon the implementation of structured design processes that amalgamate pedagogy, technology, and content knowledge, thereby ensuring congruence between learning objectives and instructional methodologies. These methodologies aspire not solely to enhance cognitive and technical competencies, but also to foster emotional, relational, and ethical development in students, who are regarded as holistic individuals who learn within inclusive and multicultural environments.

Consequently, strategic planning is imperative for the creation of educational experiences that strike a balance between authenticity, safety and practicality. Digital transformation, therefore, must transcend simplistic technological implementation and evolve towards a reorganization of teaching methodologies that are firmly anchored in pedagogical objectives, competency-based frameworks, and the sustainability of educational environments.

### Study design and instructional framework

David Kolb’s model suggests that learning is predicated upon experiential interactions [39,40], this pedagogical framework hypothesizes that learning is contingent upon experiential immersion and is structured into four distinct stages, conceptualizes learning as a cyclical process comprising concrete experience, reflective observation, abstract conceptualization, and active experimentation. Integrating these four stages fosters a dynamic relationship between practice and theory, enabling the learner to transform experience into knowledge. Consequently, a systematic approach encompassing experimentation, observation, theorization, and reﬂection is imperative to meet the proposed objectives (Table 1). However, the implementation of this process within an activity poses signiﬁcant complexity, as individuals demonstrate preferences for certain elements while avoiding others. However, if these activities are carried out with ease, profound learning outcomes can be achieved, thus fulfilling the fundamental objective of the model: that learning occurs when information is perceived and processed appropriately [40].

**Table 1:** Types of perception and forms of information processing according to Kolb.

Dimensions of learning	
Information perception	Information processing
By concrete experiences	By active experiences
By abstract conceptualization	By reﬂective observation

Source: Kolb, Honey, and Mumford’s Learning Models: For Science Education, Rodríguez (2018).

When implementing Kolb’s framework, it is essential to design activities that take into account diverse learning styles and the fundamental principles of the teaching-learning process, thereby improving academic performance and student engagement [41]. Moreover, the proposal is situated within the paradigm of design-based research (DBR) [42–44], which emphasizes iterative development, empirical testing, and refinement of educational interventions in authentic contexts. This ensures methodological robutness while producing a replicable framework that can be adapted to other STEM fields. Educational methodology based on the SEBAS framework.

It is important to consider the incorporation of the new generation of students, technological advances, and digital transformation into the higher education system, requiring motivating and guiding the objectives of the present generation of digital natives, who have been stimulated from a very early age with multimedia information [45,46], which produces anticipation of incorporating innovative teaching systems. It has been establishes that adaptive hypermedia systems (ASH) and augmented reality are the technologies that suggest a greater potential for learning processes [47,48], therefore, new technologies should favor the process of acquiring competencies as an integral model in the teaching-learning process, where knowing the context of the information would allow students to create mind maps favoring the incorporation of contents, which will require their adaptation to the type of activities, resources and tools.

The instructional intervention was designed using an instructional framework grounded in established models of technology-enhanced learning. The SEBAS methodology was informed by the Technological-Pedagogical-Content Knowledge (TPACK) framework [49,50] and the Substitution–Augmentation–Modification–Redefinition (SAMR) model [51], which guided decisions regarding the level and role of technology integration within learning activities.. These models establish progressive levels of technological integration, ranging from substitution to redefinition. The TPACK framework enables the utilization of technology as both an illustrative tool and a transformative medium, with the potential to redefine the manner in which students interact with abstract mining concepts, spatial models and operating systems.

The present study identifies AR and adaptive hypermedia systems as key drivers for contextualised and personalised learning, with recent studies confirming their potential for immersive, situated learning [52,53]. The utilization AR in the field of mining engineering offers a unique opportunity to enhance the learning experience. By employing thistechnology, students are able to visualise three-dimensional geological structures, simulate extraction processes, and make operational decisions in a safe virtual environment. This approach enables the cultivation of key competencies such as cognitive engagement, spatial reasoning, and procedural understanding, which are essential for success in the mining engineering profession.

The objective of this research is to advance the integration of augmented reality (AR) within engineering education through the development and empirical validation of a comprehensive framework for designing technology-mediated learning activities, contributes to the academic discourse in two principal dimensions. First, it presents the systematic design and preliminary validation of a pedagogical model that operationalizes AR-based learning within mining engineering education while remaining adaptable to other STEAM disciplines emphasizing its foundation on established models of technological implementation, learning methodologies, and applicability to higher education environments. Second, it contextualizes and critically examines the implementation of this technology in mining engineering, generating empirical evidence that bridges the current gap between emerging educational technologies and discipline-specific learning practices.

Given the educational context and the minimal risk involved in the study, consent was obtained verbally from the students. The lead researcher verbally explained the study using an approved information consent form to ensure that students understood the study’s purpose, procedures, and benefits, as well as their rights.

With the student’s permission, a second researcher served as a witness to validate the consent and confirmed that the participants had understood and voluntarily agreed to participate. No identifying information was linked to survey responses. Moreover, the universities involved have a service for academic training and awareness of personal data protection processing, and the relevant rules and guidelines were strictly followed by all researchers involved in this study. Additionally, the faculty overseeing the study are IRB-trained.

### Materials and resources

The study addressed the incorporation of AR into higher education for courses in mining and metallurgy related programs. Initially, information on the implementation of AR in teaching was collected and the state of the art was studied to understand the advantages, disadvantages, and requirements of this technology. Challenges were faced in the use and management of three-dimensional (3D) modeling tools and software programming, which led to the search for cost-eﬃciency alternatives. For the proposals concerning the development of activity modeling tools, the BLENDER program was utilized alongside block programming for the generation of internal code through the COSPACE/DELIGHTEX platform, thereby facilitating the effective formulation of these activities. To this end, priority was given to students who had the bases and previous knowledge of the subjects that would be addressed in the implementation of the tests, developing more than 15 of their own digital resources and a library of at least 40 freely accessible modeled virtual objects collected from web libraries, with a free license for the target subjects. In addition, prior to the execution and development, they had the opportunity to work with students of advanced courses of the specialty, being able to validate their capacity of previously acquired competencies and showing a high level of participation in the development process, where, not only did they work for an established objective among their tasks, but they were also able to strengthen the identiﬁcation, characterization and application of concepts and technical knowledge for the elaboration of 3D models generating standard documents to facilitate the use of their models in a training context.

### Data collection instrument

In consideration of the prevailing educational context and the ethical and logistical constraints associated with random assignment in higher education settings, a quasi-experimental design with a non-equivalent control group was adopted [54,55]. The design of the study enables the comparison of learning outcomes and motivational indicators between two groups of participants. One group is exposed to a SEBAS-based Augmented Reality (AR) learning intervention, while the other follows the traditional teaching approach. Despite the unfeasibility of randomization, equivalence between groups was established through the selection of groups enrolled in the same academic courses, exhibiting comparable demographic and academic characteristics.

This methodological decision is consistent with the recommendations for applied educational research, as it allows for the examination of causal relationships between instructional interventions and learning outcomes while maintaining internal validity. First, quantitative historical assessments establish a baseline for academic performance. This approach allowed for an objective and statistically signiﬁcant comparison of the results obtained before and after the implementation of the technology [56], in addition, qualitative surveys were used both before and after the workshops.

The pre-surveys were designed to capture students’ initial expectations and knowledge about augmented reality, while the exit surveys focused on measuring perceptions, motivation and perceived impact after the educational intervention. This qualitative approach provided a deep and holistic understanding of the students’ experiences, complementing the quantitative data historical assessments and surveys were designed and administered following standardized protocols to ensure the validity and reliability of the data collected [57].

To determine whether there were any statistically significant differences in academic performance between the intervention group and the comparison group, a Welch’s t-test was performed(20) [58]. This inferential statistical test was chosen specifically due to its robustness and suitability for comparing means when the assumption of homoscedasticity (equal variances) cannot be met a common occurrence in quasi-experimental designs.

### Procedure for creating and implementing AR activities

Table 2 illustrates the six-week sequential implementation of the SEBAS methodological framework within the ‘Mining Operations and Equipment’ course. Five weeks are defined for the presentation and experimentation with the study group, each representing the execution of a specific stage of the model (selection, establishment, blueprinting, application, supervision and synthesis). This corresponds to a structured progression of design, execution and evaluation activities (the former being the stage at which the pre-test is taken and the latter being the stage at which the post-test is administered). This provides a replicable reference for other similar AR-based educational interventions.

**Table 2:** Sequential implementation of the SEBAS methodological framework for integration in engineering education.

Week 1	Week 2	Week 3	Week 4	Week 5–6
Selection	Establishment	Blueprint	Application	Synthesis and Supervision (S)
Concept & Context	Technical Setup	Prototype Design	Implementation	Evaluation & Reflection
•Define learning objectives and competencies. •Identify mining operations (e.g., drilling, hauling). •Select appropriate software tools (Blender, CoSpaces).	• Specify AR tools and technical parameters. • Align AR content with curriculum objectives. •Co-create 3D resources and content structures.	• Develop and test interactive AR prototypes. •Conduct usability walkthroughs. • Peer review of AR models and user interfaces.	•Deploy finalized AR activities in lectures and labs. • Collect quantitative and qualitative data. •Evaluate student engagement and motivation.	• Analyze results and reflect on learning outcomes. •Collect feedback from students and instructors. •Refine the SEBAS framework for future replication.

***Source: Authors’s Creation***

SEBAS Framework The SEBAS methodology is grounded in the principles of Design-Based Research used to engineering education (DBR), emphasizing iterative cycles of design, implementation, evaluation, and refinement in authentic educational settings, aligning with Kolb’s experiential learning cycle and integrating concepts from TPACK and SAMR, resulting in a new development framework for activities integrating AR into the design process.

### Selection (S)

The selection phase formed the conceptual basis of the SEBAS model. The instructional team focused on defining the pedagogical intent, scope and relevance of AR integration. They identified the learning outcomes, target competencies and operational contexts in which AR could specifically enhance conceptual and procedural understanding within the topics of drilling, loading and hauling equipment.

**Fig 3:**
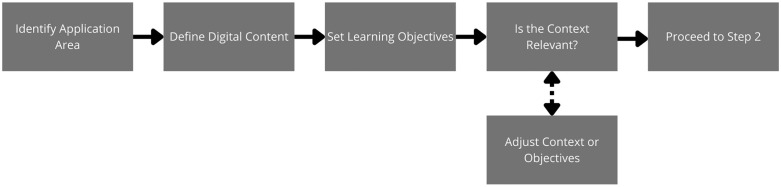
Workflow for selecting content and context in AR learning environments.

This involved reviewing the course objectives and ensuring that the introduction of AR was pedagogically justified, as well as aligning the curriculum. The selection of content and technological tools (Blender and CoSpaces Edu) was based on educational needs and feasibility criteria. The outcome of this phase was a validated pedagogical map that defined which learning experiences would be augmented, why they had been selected and how they aligned with the expected learning outcomes (see Fig 3).

### Establishment (E)

The objective was to transform the conceptual blueprint into a structured design plan by specifying the necessary tools, parameters and development requirements. The team established content accuracy standards, defined 3D modelling requirements and determined key variables, such as scale, level of interactivity and visual complexity.

**Fig 4:**
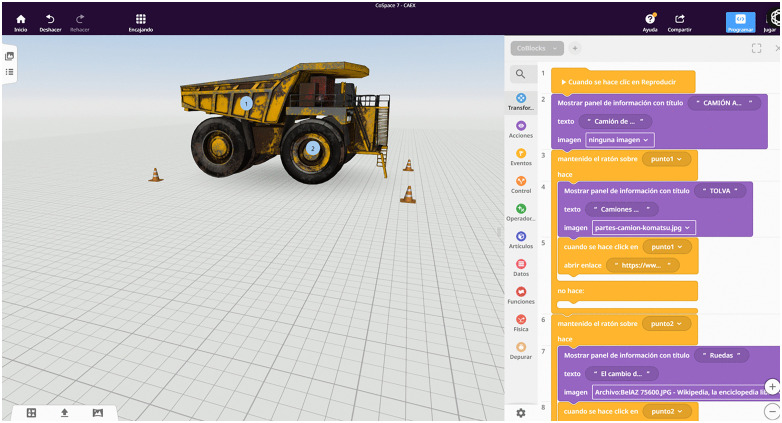
Interactive 3D model of a haul truck developed in Delightex/CoSpaces Edu.

Activities at this stage included collaboratively designing 3D representations of mining equipment (e.g., shovels, haul trucks, and drilling rigs) and defining interactive markers and user triggers within the AR environment (Fig 4). Each prototype element was cross-checked against the course syllabus to ensure alignment with the competencies defined in Week 1. This phase resulted in a technical design dossier that formed the basis for the creation of subsequent prototypes and pedagogical validation.

### Blueprint (B)

This phase primarily functions as a design validation cycle, bridging the gap between conceptual design and empirical testing. It seeks to transform theoretical plans into functional prototypes through iterative, evidence-based design that reflects the principles of design-based research (DBR).

**Fig 5:**
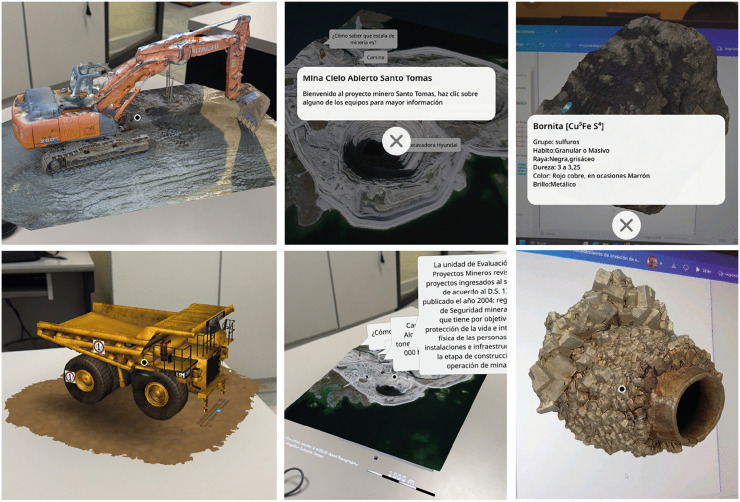
Illustrations of augmented reality activities utilizing the SEBAS methodology.

Low- to high-fidelity prototypes are developed to simulate operational processes within the AR environment. Activity instructors and some student assistants (i.e., students who have previously passed the course) are involved in interacting with the prototypes and conducting usability walks, interface evaluations and scenario calibration. This ensures intuitive navigation and balanced cognitive load, both of which are important considerations for the development of adaptive hypermedia activities. For the formulated scenario in the experiment, missing 3D assets were generated using Blender and integrated into the scenario in CoSpaces Edu to maintain harmony between realism, interactivity and pedagogical relevance. You can see some of the implemented models and activities in Fig 5 of the case study.

### Application (A)

**Fig 6:**
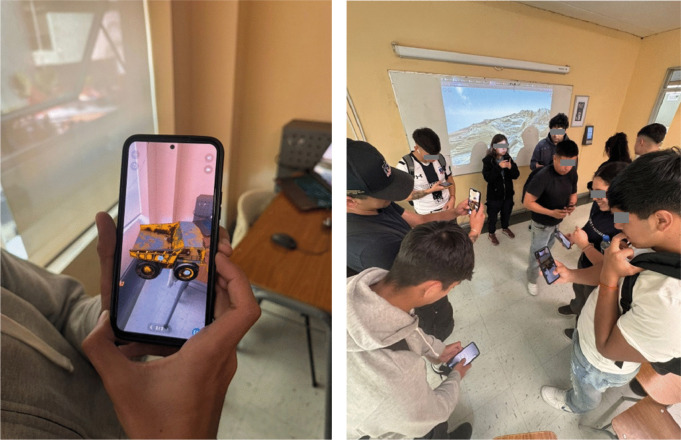
Implementation of SEBAS-based learning experiences in authentic environments.

This stage involves the implementation of learning experiences in the classroom setting, the empirical execution of activities (as illustrated in Fig 6) designed by the SEBAS development framework within authentic learning environments, and the subsequent evaluation of their pedagogical impact.

The activities were conducted through two distinct implementation sessions, spaced a week apart, in which the subject of mining operations and mining equipment was presented. Prior to each session, a class on theoretical concepts was held, following the methodological processes for the correct implementation of the technology and the achievement of the expected learning outcomes, with these practical laboratory activities using AR simulations. The students engaged with three-dimensional models of mining equipment to visualize mechanical cycles, perform operational sequences, and analyze safety constraints. These activities were designed to encourage exploration, hypothesis formulation, content integration, and reflection (see Table 3).

**Table 3:** Evaluation components and learning dimensions integrated activity design.

Knowledge acquisition	Practical application	Reflective learning
Pre- and post-activity questionnaires to assess conceptual improvement, perception, and motivation.	AR-based simulations to assess understanding of procedures	Guided prompts that encouraged students to connect virtual tasks with real operating systems.

*Source: Author’s creation.*

In the present study, quantitative and qualitative data were triangulated through academic performance records, participation metrics, and perception surveys, ensuring internal validity and providing multidimensional evidence of learning effectiveness and user experience.

### Synthesis and supervision (S)

The subsequent stage focused on analysing the data collected, synthesising the conclusions drawn from both quantitative performance measures and qualitative comments, and integrating improvements into the pedagogical and technical design. The aim of these feedback loops between teachers and students was to encourage iterative reflection, reinforcing the principles of continuous improvement inherent in DBR and updating the presentation of activities.

The evaluation process verified the alignment between learning outcomes, AR activities, and student performance indicators, ensuring that pedagogical intentions were effectively translated into measurable results. The final synthesis resulted in a revised version of the SEBAS framework, incorporating recommendations for scalability and adaptability to other engineering disciplines.

### Proposal methodology for designing AR activities

**Fig 7:**
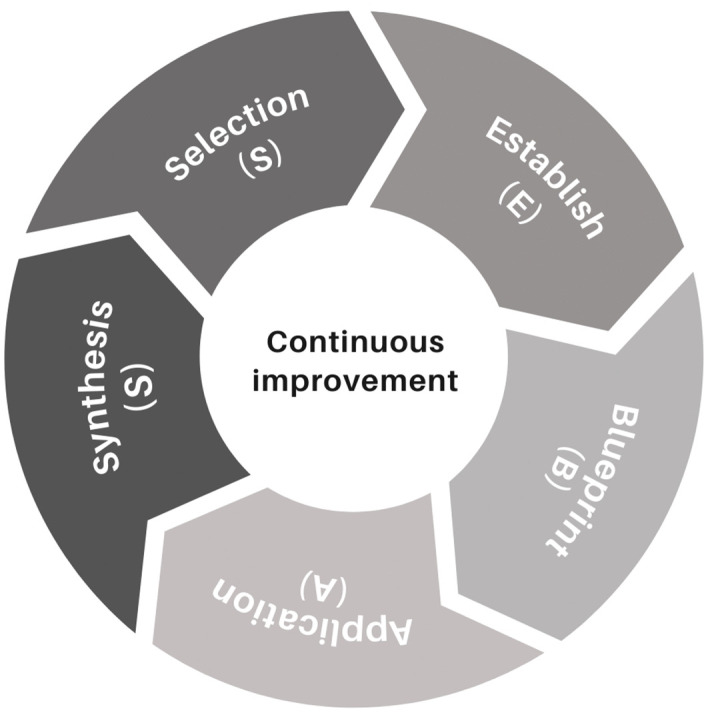
Circular diagram of the SEBAS methodology. Diagram created by authors; no copyright restrictions.

The concluding phase entails the evaluation of the components, activities, and interactions within the implemented development, coupled with adjustments based on targeted feedback and impact assessments. This phase should encompass not only the collection of such feedback but also a thorough evaluation to validate the anticipated content, knowledge, and learning outcomes [Fig 7]. This process is crucial for integrating lessons learned and recommendations, thereby facilitating the creation of an enhanced iteration of the project if required, while ensuring its congruence with the initial objectives and the needs of the students.

**Fig 8:**
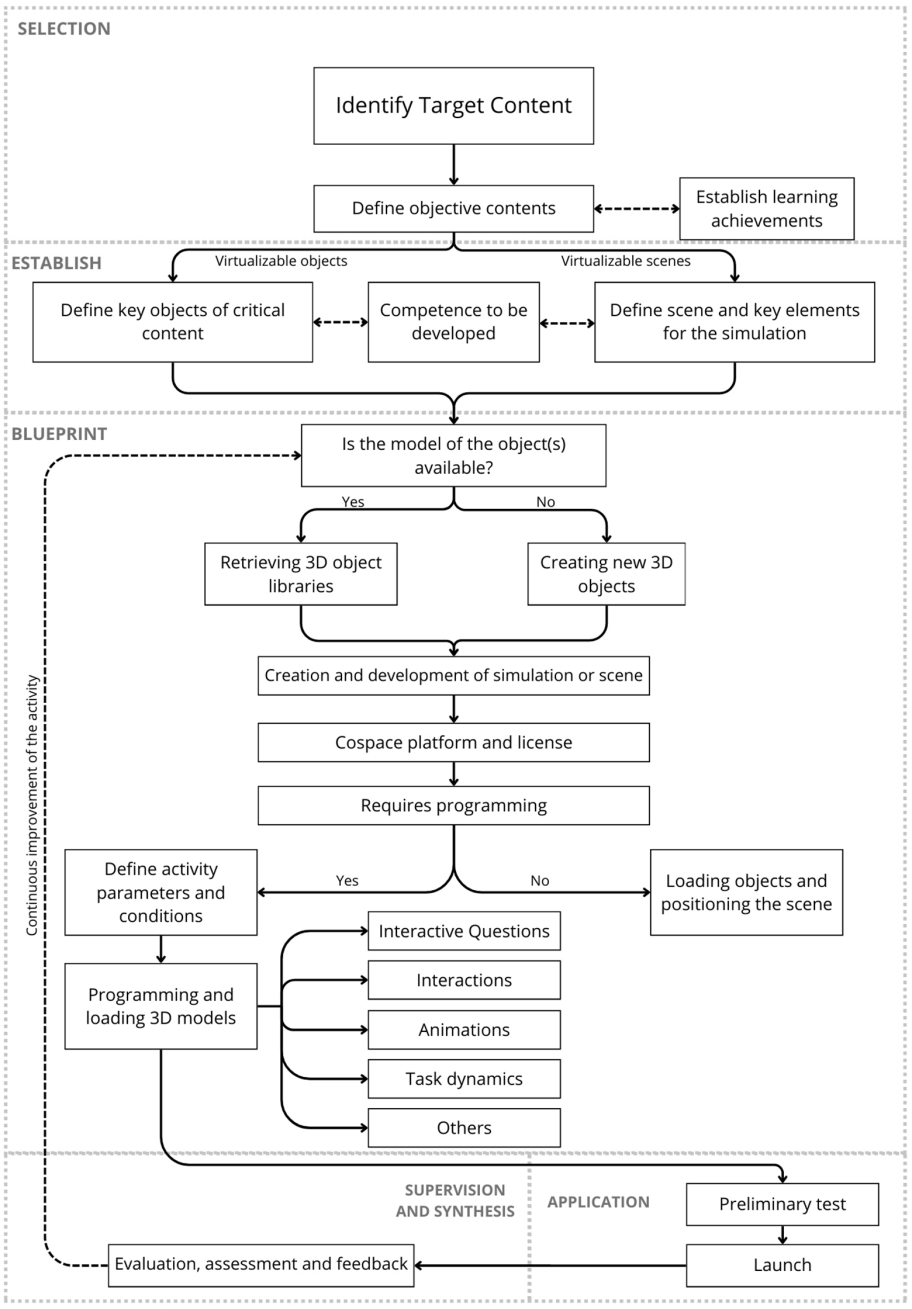
Comprehensive methodology for AR activities. Diagram created by authors; no copyright restrictions.

The diagram to Fig 8 details a comprehensive methodology designed to develop and implement educational activities using AR, facilitating a systematic approach that ranges from content selection to implementation and ﬁnal review. It begins with the selection of appropriate subjects and the deﬁnition of clear educational objectives, followed by the speciﬁcation of technical and content requirements, proceeding to the sketching stage for the design and development of the activity requirements, the process moves forward as these models are integrated into interactive scenarios that are tested and adjusted based on direct feedback, culminating in supervision and synthesis where the effectiveness of the implementation is evaluated and the necessary adjustments are made, this allows the process not only to ensure that the resources are pedagogically effective, but also to adapt the technology to the speciﬁc needs of the educational environment, maximizing the impact on student learning and providing an immersive and enriching experience.

## Results

As mentioned previously, pre-intervention pre-tests were used to capture students’ initial expectations and prior knowledge of AR, while post-test surveys focused on changes in perceptions, motivation, and perceived educational impact following the intervention. This qualitative data complements the quantitative indicators of academic performance and supports a triangulated analysis of educational outcomes.

As shown in Table 4, historical academic records were incorporated as a quantitative reference to contextualize the outcomes of the SEBAS-based AR intervention. The pass rate increased from 57% in 2022 to 68% in 2023, and the overall grade point average showed a modest upward trend, rising from 4.0 to 4.31 on a 1.0–7.0 grading scale. These descriptive trends suggest an improvement in academic outcomes across cohorts; however, given the quasi-experimental design, they should be interpreted as contextual indicators rather than causal effects

**Table 4:** Historical assessments by subject period.

Row Labels	Final grade average	Sum of quantity	APR/REP Percentage
**2022**			
**APPROVED**	5.9	8	57%
**REPROACHED**	1.4	6	43%
**Total**	4.0	14	
**2023**			
**APPROVED**	5.53	93	68%
**REPROACHED**	1.67	43	32%
**Total**	4.31	136	

*Source: Authors’s Creation*

Historical data were therefore not analysed in isolation, but rather served as a quantitative reference to reinforce triangulation with students’ self-reported perceptions, motivation and satisfaction, which were collected through pre- and post-intervention surveys.

According to the results presented in Table 5, the 136 student population who participated in the five 2023 courses achieved an average final grade of 4.31, with a standard deviation of 2.16. The overall grade distribution exhibited more concentrated behaviour among students who participated in the Augmented Reality (AR) workshops, characterised by a lower standard deviation (1.75) than the 2023 group as a whole (2.16). This reduction in variability suggests more consistent academic performance within the AR group, potentially reflecting greater engagement and comprehension through immersive learning activities.

**Table 5:** Results of the evaluations.

Variable	2022 No workshop	2023 global	2023 with workshop
**Count**	14	136	74
**Average**	3.96	4.31	4.70
**Desv. Standard**	2.43	2.16	1.75
**25%**	1.00	2.70	4.50
**50%**	5.10	4.95	5.00
**75%**	6.00	5.92	6.00
**Max.**	6.70	6.90	6.90

*Source: Author’s creation.*

Examining performance trends across AR workshops revealed that participants consistently achieved higher and more consistent grades, suggesting a positive impact on learning outcomes. For example, the 25th percentile for the AR group was 4.50, which is notably higher than the same percentile for the non-AR group (2.70). This suggests that even the lowest-performing students in AR-based classes achieved better results. These findings support the hypothesis that integrating AR into teaching fosters a deeper understanding of complex engineering concepts and improves academic achievement. Table 5 summarises the aggregated results for 2022 (excluding workshops), the 2023 group as a whole and the 2023 group that participated in AR activities. The data reveal a steady improvement in performance, rising from an average of 3.96 in 2022 to 4.70 in 2023 for the group that participated in AR activities, accompanied by a decrease in dispersion from 2.43 to 1.75. This further supports the consistent performance observed in AR-integrated courses.

A more detailed analysis was conducted across the five courses included in the 2023 academic year. As shown in Table 6, the results vary slightly between courses, reflecting differences in teaching strategies and content areas.

**Table 6:** Disaggregated performance results by course.

Course	Variable	2023 with workshop	2023 No workshop
**1**	Count	14	25
	Average	4.76	3.20
	Desv. Standard	0.96	1.99
**2**	Count	39	3
	Average	4.51	1
	Desv. Standard	2.19	0
**3**	Count	7	5
	Average	4.60	4.40
	Desv. Standard	0.78	2.93
**4**	Count	14	2
	Average	5.2	3.05
	Desv. Standard	1.22	2.05
**5**	Count	27	
	Average	4.7	
	Desv. Standard	2.34	

*Source: Author’s creation.*

Statistical analyses using Welch’s t-tests revealed statistically significant differences in Courses 1 and 2 (p < 0.01), with students who participated in the AR workshops achieving notably higher final grades than their peers with no AR exposure. Although the differences were not statistically significant in Courses 3 and 4, the mean grades for the AR groups remained equal to or higher than those of the non-AR groups. Course 5, which did not implement AR, served as a general control group, displaying comparable averages but greater variability. This suggests that the positive effect of AR depends on the degree of methodological integration and the nature of the practical activities carried out.

These findings demonstrate the importance of integrating technology to improve academic performance and consistency, especially when it is effectively incorporated into an active, practice-oriented pedagogical framework.

The initial survey on AR expectations and prior knowledge reveals a split in previous AR experience, with a low of students having had exposure to this technology. However, conﬁdence in its ability to improve understanding of exploitation methods is relatively low, suggesting skepticism about its practical applicability, noting that one-ﬁfth of respondents consider that implementing AR would be diﬃcult in their training (Table 7).

**Table 7:** Analysis of qualitative surveys by macro-dimensions.

Variable	Answer	Percentage (%)
**Previous experience**	Yes	30.6
	No	69.4
**Knowledge or perception of AR use**	Yes	37.2
	No	62.8
**Degree of implementation complexity**	Easy	77.6
	Diﬃcult	22.4

*Source: Authors’s Creation*

The results of the ﬁnal (Table 8) survey show an increase in satisfaction among students who participated in AR activities in their training. 60.5% of respondents expressed a high degree of satisfaction, while only 2.3% said they felt dissatisﬁed. Likewise, the vast majority consider that the skills acquired are applicable to practical contexts, highlighting the usefulness of AR in technical education.

**Table 8:** Qualitative 3D output survey analysis.

Variable	Answer	Percentage (%)
**Satisfaction with AR**	Very satisﬁed	60.5
	Satisﬁed	16.3
	Neutral	14.0
	Unsatisfied	7.0
	Very Unsatisfied	2.3
**Applicability of acquired skills**	Practical	92.9
	Not applicable	7.1

*Source: Authors’s Creation*

When comparing the input and exit surveys of the workshop activities, a positive change is observed in the students’ perception of usefulness, satisfaction, as well as conﬁdence that it can improve the understanding of the exploitation methods. In addition, most students now ﬁnd the implementation of AR to be easy and applicable to their hands-on training.

The combined dataset includes students’ grades and their responses from entry and exit surveys (Table 9), providing a comprehensive view of how their acceptance of AR correlates with their performance

**Table 9:** Comparison of qualitative results.

Variable	Input (%)	Output (%)
Previous experience with AR	69.4	–
Satisfaction with AR	–	60.5
Conﬁdence in improvement with AR	37.2	84.1
Ease of Deployment	77.6	90.7
Applicability of skills	–	92.9

*Source: Authors’s Creation*

In the initial acceptance level, it is observed that the students rated their acceptance of the AR as high, tending to maintain or improve their performance in the general grades of the subject of the workshop activities, on the other hand, the initially undecided students showed better levels of acceptance after the practical presentation in the workshops.

## Discussion

The results of this study provide evidence that the strategic integration of Augmented Reality (AR) within engineering education can positively influence both academic performance and student engagement, particularly when supported by a structured pedagogical framework such as SEBAS. Rather than functioning as a standalone technological enhancement, AR demonstrated its educational value when aligned with clear learning objectives, experiential learning principles, and active instructional strategies.

**Fig 9:**
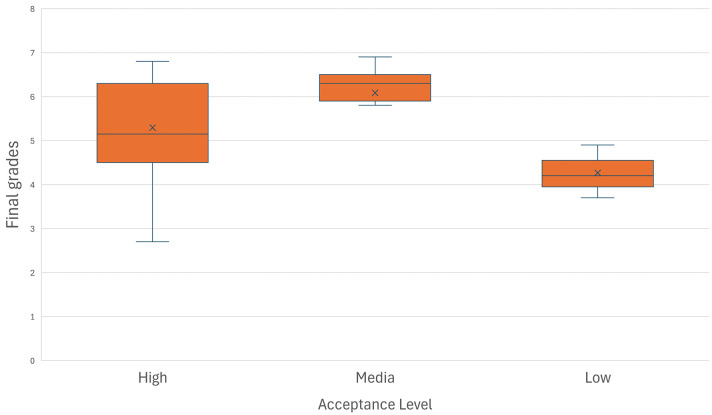
Distribution of acceptance levels based on grade among participants.

Fig 9 provides additional insight into the relationship between students’ initial acceptance of AR technology and their academic performance. A discernible pattern emerges whereby students who possessed with a medium or high initial acceptance generally obtained better final grades. There is a general trend of increasing average grades with higher initial acceptance, facilitating the visualization of how ﬁnal grades ﬂuctuate in relation to students acceptance levels of AR technology, thereby offering a coherent perspective on the inﬂuence of this technology on academic performance. Moreover, the reduced variability in performance among students with higher acceptance levels points to a stabilizing effect of immersive learning experiences, reinforcing consistency in learning outcomes across diverse student profiles.

The findings suggest that the strategic amalgamation of AR pedagogical activities, designed and progressively refined through a systematic methodological framework anchored in instructional design tenets, correlates with enhanced academic achievement and a diminishment of variability in student outcomes. This uniformity in performance implies that such interventions may foster a more equitable educational experience and better equip students to confront authentic industrial challenges. In order to maintain their pedagogical efficacy, AR-based activities necessitate ongoing updates concerning content, instructional methodologies, interactive and participatory approaches, evaluation systems, as well as requisite periodic assessments and validation to confirm their sustained relevance and adaptability in light of shifting cultural, social, and technological paradigms [59], this is consistent with recent meta-analytical evidence indicating that AR’s medium-to-high effect size on learning gains is most pronounced when activities are integrated into collaborative and problem-based settings [60]

Importantly, these results emphasize that the educational impact of AR is not driven by technological novelty alone, but by its purposeful integration within a structured instructional framework [61,62]. The SEBAS methodology operationalizes this integration by ensuring coherence between pedagogical intent, technological design, and assessment strategies through iterative refinement. In doing so, it addresses a critical gap identified in the literature, where many AR applications lack transferability due to insufficient pedagogical structure [63].

However, the optimization of the educational efficacy of augmented reality (AR) necessitates meticulous consideration of pedagogical and cognitive parameters. The construction of AR learning artifacts must encompass a variety of learning modalities, preferences for interaction, and limitations regarding cognitive load to guarantee accessibility and substantive learning experiences for all learners [64]. Prior investigations have illustrated that inadequately formulated immersive environments can inundate students and impede educational outcomes if the intricacies and usability of instructional frameworks are not appropriately regulated [65,66]. As a result, ongoing evaluation and iterative enhancement of AR interventions are imperative to sustain their effectiveness and pertinence. Subsequent inquiries should explore longitudinal implementations, conduct multi-institutional studies, incorporate psychosocial factors, and amalgamate augmented reality with complementary instruments such as learning analytics or artificial intelligence to enhance personalization and feedback mechanisms.

## Conclusion

This study examined the integration of augmented reality in mining engineering education through the design and validation of the SEBAS methodological framework. Using a mixed-method, quasi-experimental approach in authentic educational contexts, the findings indicate that strategically designed AR-based learning activities are associated with improved academic performance and reduced variability in student outcomes, particularly in courses with higher levels of methodological integration. The highlight that educational impact depends not on the technology itself, but on its alignment with instructional design principles, learning objectives, and active-participatory methodologies. Complementary qualitative evidence shows a positive shift in students’ perceptions, motivation, and perceived applicability of acquired skills, suggesting that well-designed immersive experiences can mitigate initial skepticism and enhance engagement with complex technical content.

The main contribution of this research is the SEBAS framework, which provides a structured, pedagogically grounded, and replicable approach for integrating augmented reality into engineering education. While developed in a mining engineering context, the framework is adaptable to other engineering and applied science disciplines. Future research should explore longitudinal implementations and broader disciplinary applications to further validate and extend the framework’s effectiveness.
